# Extensive Epidural Cerebrospinal Fluid Collection With Cauda Equina Compression Following Lumbar Puncture

**DOI:** 10.51894/001c.165583

**Published:** 2026-07-31

**Authors:** Mckalin Cox, Michael Collins, David Moore

**Affiliations:** 1 Henry Ford - Wyandotte

## 74

### Introduction

Lumbar puncture (LP) is a routine diagnostic procedure most commonly complicated by post-dural puncture headache. Extensive epidural cerebrospinal fluid (CSF) collections producing neurologic deficits are rare.

### Case Presentation

A 25-year-old female underwent an uncomplicated diagnostic lumbar puncture during evaluation for suspected multiple sclerosis. Approximately 24 hours later, she developed severe positional headache, back pain, nausea, vomiting, and urinary retention. Examination revealed diminished rectal tone without saddle anesthesia. MRI of the spine demonstrated extensive posterior epidural CSF extending from C6–C7 through the thoracic and lumbar spine to the sacral canal, as well as anterior epidural fluid from T12–L1 to the sacrum. The collections were T1 hypointense and T2 hyperintense, consistent with CSF, and produced significant thecal sac narrowing with crowding of the cauda equina nerve roots. There was no evidence of epidural hematoma or abscess.

### Management

After excluding compressive hematoma or infection, a targeted epidural blood patch was performed. The patient experienced rapid improvement in headache and back pressure, with gradual resolution of urinary retention over 12–24 hours. Repeat neurologic examination demonstrated normalization of neurological deficits.

### Discussion

Post-lumbar puncture headache typically results from intracranial hypotension rather than spinal mass effect. Rarely, persistent CSF leakage may dissect extensively through the epidural space, accumulating across multiple spinal levels and producing symptoms that mimic cauda equina syndrome. Bladder dysfunction following LP is unusual and should prompt urgent spinal imaging. MRI is essential to differentiate CSF collections from epidural hematoma or abscess, as management strategies differ substantially. Blind epidural blood patch without imaging may risk worsening neurologic compression in these alternative diagnoses.

### Conclusion

Extensive epidural CSF collections following lumbar puncture can rarely produce cauda equina compression. Clinicians should obtain urgent MRI when urinary retention or neurologic deficits occur after LP. Early recognition and targeted epidural blood patch result in favorable outcomes.

